# Impact of prenatal maternal psychological distress on fetal biometric parameters in household air pollution-exposed Nigerian women

**DOI:** 10.1371/journal.pone.0272053

**Published:** 2022-07-28

**Authors:** Oluwafunmilade Deji-Abiodun, Babatunde Adedokun, Donee Alexander, Anindita Dutta, Tope Ibigbami, John Olamijulo, Dayo Adepoju, Samuel Adekunle, Oladosu Ojengbede, Christopher O. Olopade

**Affiliations:** 1 Department of Medicine and Center for Global Health, University of Chicago, Chicago, IL, United States of America; 2 Healthy Life for All Foundation, University College Hospital, Ibadan, Nigeria; 3 Department of Obstetrics and Gynecology, University of Ibadan, Ibadan, Nigeria; Anton de Kom Universiteit van Suriname Faculteit der Medische Wetenschappen, SURINAME

## Abstract

**Rationale:**

Studies identify prenatal household air pollution (HAP) exposure and maternal psychological distress (PMPD) as independent factors contributing to gestational ill-health and adverse birth outcomes.

**Objective:**

We investigated the impact of PMPD on fetal biometric parameters (FBP) in HAP-exposed pregnant Nigerian women.

**Methods:**

The randomized controlled trial (RCT; ClinicalTrials.gov NCT02394574) investigated effects of HAP exposure in pregnant Nigerian women (n = 324), who customarily cooked with polluting fuels (firewood or kerosene). Half of the women (intervention group) were given CleanCook ethanol stoves to use for 156 days during the study. Once a month, all women were administered an abridged version of the SF-12v2^TM^ health-related quality of life questionnaire to assess psychological distress. Using mixed effects linear regression models, adjusted for relevant covariates, we analyzed associations between the women’s exposure to PM_2·5_ (particulate matter with an aerodynamic diameter<2_·_5 microns) from HAP, their PMPD scores, and FBP (ultrasound estimated fetal weight [UEFW], head circumference [HC], abdominal circumference [AC], femur length [FL], biparietal diameter [BPD], estimated gestational age [GA] and intrauterine growth restriction [IUGR]), and birth anthropometric measures (birth weight [BW] and birth length [BL]).

**Results:**

PMPD negatively impacted UEFW, HC, FL, BPD and BL (p<0_·_05). Controls (kerosene/firewood users) experienced significantly higher PMPD compared with ethanol-stove users (p<0_·_05). The mediation analysis revealed that the proportion of the outcome (fetal biometrics, birth anthropometrics, IUGR and GA), which can be explained via PMPD by groups (intervention vs. control) after adjusting for confounding variables was 6_·_2% (0_·_062). No significant correlation was observed between levels of PM_2.5_ exposure and PMPD scores.

**Conclusions:**

PMPD was an independent mediator of adverse fetal biometric parameters in pregnant women, who were exposed to HAP from burning of firewood/kerosene. Formulating preventative measures to alleviate maternal distress during pregnancy and reducing exposure to HAP is important from public health perspectives.

## Introduction

Globally, about one billion people were affected with mental illness in 2016. This was estimated to be 7% of the global burden of disease, as measured in disability-adjusted life-years [1]. Mental health deterioration, more prevalent among women than men, has profound adverse effects on cardiovascular health and birth outcomes [2]. Women’s mental health remains a neglected area of medical attention, especially in low- and middle-income countries (LMICs). To bridge the gaps in our knowledge of women’s mental health in LMICs, it is necessary to undertake more research [3]. Additionally, household air pollution (HAP) resulting from cooking with unclean fuels is a pervasive problem in LMICs [4] and has been shown to increase the risk of depression in pre-menopausal rural Indian women [5]. Though studies have independently linked exposure to air pollution with psychiatric disorders [6], depression, anxiety, prenatal maternal distress [5,7,8] and adverse birth outcomes [9,10] those exploring association of maternal distress during pregnancy with fetal and birth outcomes in an air pollution exposure setting are limited. Lin and colleagues observed increased risks of prenatal maternal psychological stress with increasing levels of various ambient air pollutants [11]. However, they did not explore potential associations between prenatal stress and any adverse effects on fetal biometrics. Ae-Ngibise K et al. found that high prenatal maternal stress was associated with adverse birth outcomes among rural Ghanaian women participating in the Ghana Randomized Air Pollution and Health Study (GRAPHS) [12]. However, they did not leverage GRAPHS data to investigate air pollution exposure and resulting maternal distress as possible combined factors affecting the observed birth outcomes.

The association of prenatal maternal psychological distress (PMPD) with fetal and birth outcomes is under-studied in sub-Saharan Africa [13]. In order to address this shortage of research and to explore HAP as a possible contributing factor, we undertook this study, which is ancillary to a completed, randomized controlled trial (RCT) in Nigeria. We evaluated the impact of PMPD on fetal biometric parameters [FBP; ultrasound estimated fetal weight (UEFW), head circumference (HC), abdominal circumference (AC), femur length (FL), biparietal diameter (BPD) and intrauterine growth restriction (IUGR)], birth weight (BW), birth length (BL) and estimated gestational age (GA) in a RCT evaluating the effects of HAP exposure on pregnant women, their fetuses, and newborns. For this ancillary study, the primary hypothesis was that women with higher PMPD scores would show poorer FBP outcomes than women with lower scores. The secondary hypothesis was that women cooking with firewood or kerosene (the control group) would have higher PMPD scores compared with ethanol users (the intervention group).

## Materials and methods

### Study design, eligibility criteria and participants

We examined our hypotheses concerning the impact of PMPD on fetal biometric parameters by leveraging the parent RCT, which was conducted in Ibadan, Nigeria from June 2013 to October 2015. The details of subject recruitment have been discussed in our earlier publications [9,10]. Briefly, the parent study enrolled 324 apparently healthy women, who were their household’s primary cook, less than 18 weeks pregnant, and used wood and/or kerosene as their primary cooking fuel. Women were excluded if they were HIV positive, smokers, lived with a smoker, cooked for a living, or had a high-risk pregnancy (multiple gestations, uncontrolled maternal hypertension, older than 35 years at first delivery, three or more miscarriages, or previous cesarean section). All evaluations of fetal biometric parameters were made simultaneously at the same gestational age. Eligible women, who agreed to participate were recruited into the study and signed written informed consent forms. We randomized each pregnant women to the intervention or control arm of the study. The intervention group were given CleanCook ethanol stoves (CLEANCOOK Sweden AB) and were supplied with ethanol fuel to use for 156 days during the study. The control group continued using firewood/kerosene as cooking fuel. The original RCT was powered to detect a difference of 250 g in birthweight between the intervention and control groups at 80% power and 5% level of significance. The Institutional Review Boards (IRB) of the University of Ibadan and the University of Chicago approved the study protocol. Participants who were less than 18 years of age had either their husband or a parent sign the consent form along with them as part of the consent process. This current ancillary study is part of a larger randomized control trial, which is registered with ClinicalTrials.gov (NCT02394574) [9,10].

### Personal exposure monitoring of PM_2.5_

We used RTI MicroPEMs to assess levels of personal PM_2.5_ exposure of each woman over a period of three consecutive days (72-hours) at two time points: once during the second trimester and once during the third trimester of pregnancy. Details of measurement procedures with the RTI MicroPEM have been provided in our earlier publications [9,10]. The mean PM_2·5_ across both measurement intervals was used in the analyses.

### Assessment of psychological distress

We used an abridged version of the Medical Outcomes Study Short Form 12 Health Survey, version 2 (SF-12v2^TM^) Health-related Quality of Life (HRQOL) to determine the pregnant women’s psychological distress levels [13,14]. The SF-12v2^TM^ is Likert scale-based HRQOL questionnaire [13] which assesses an individual’s psychological and physical health over time [15]. Based on our understanding of the cultural, emotional, and ethical needs of the study participants, we pared down the standard 12-question SF-12v2^TM^ questionnaire to seven questions. Trained study team members, who are native bilingual (Yoruba and English) speakers as well as trained linguists, translated the abridged questionnaire to Yoruba, one of the official languages in Nigeria and the dominant regional language in southwestern Nigeria, where the study was conducted. As a quality control measure, the Yoruba version was back translated into English. Then, the original and back-translated versions were compared. Any discrepancies were resolved in discussion among team members. We verbally administered the modified questionnaire to each study participant on a monthly basis during the study.

### Fetal biometric parameters and birth anthropometric measures

The biometric parameters assessed were (UEFW), (HC), (AC) (FL), (BPD), (IUGR), (BW), (BL), and (GA). A dedicated and trained radiologist blinded to the groupings determined fetal biometric parameters; including (UEFW), (HC), (AC), (FL) and (BPD) 6 times on each pregnant woman using a portable Sonosite Micromaxx ultrasound system (Bothell, WA, USA). Fetal weight was estimated using the Hadlock method [16]. Presence of IUGR and estimated GA were also recorded. As a quality control measure, a consultant Radiologist who was also blinded to the randomization grouping independently reviewed the biometric parameters and discussed the findings with the primary radiologist. The final readings reflect agreement on the biometric values. We determined birth weight (BW) and birth length (BL) immediately after delivery using a Delecto Digital baby scale (Webb City, MO).

### Statistical analysis

Associations of PMPD with UEFW, HC, AC, FL, BPD, BW, BL, mean 72-hour PM_2·5_ exposures, IUGR and GA at birth were investigated. A two-stage score aggregate was done on each of the seven questions. We used the numerical Likert scale for each of the items of the questionnaire to compute a maximum distress score of 27. After completing the questionnaire and scoring the responses, we summed up the scores, where higher scores indicated higher levels of PMPD. All birth outcomes were in quantitative units. Mixed effects linear regression models were used to evaluate bivariate associations between mean 72-hour PM_2·5_, groups (control vs. intervention), PMPD scores, fetal biometric parameters (UEFW, HC, AC, FL, BPD), birth anthropometric measures (BW, BL), IUGR and GA at birth. The random intercept models used the simple exchangeable covariance structure. Models were adjusted for various covariates, including maternal age, number of children, educational level, marital status, child’s sex, body mass index, serum biomarkers of nutritional status (folic acid, albumin, prealbumin, retinol binding protein), mother’s malaria status, frequency of cooking, and kitchen location. Mediation analysis was done to determine the degree of association between PM_2·5_ exposure levels, groups (intervention vs. control), fetal biometric parameters and birth anthropometric measures explained by distress score [17]. STATA paramed option was used [18]. We calculated the proportion of outcome mediated as the ratio of natural indirect effect to the total marginal effect. Statistical significance was set at p < 0_·_05. We used STATA statistical software, release 14 to perform these analyses [19].

## Results

Of the 324 pregnant women enrolled in the RCT (Table 1), complete data for PMPD analysis were available for 281 women (86_·_7%). Data for the remaining 13_·_3% women were excluded for various reasons, including relocation outside the study area, quitting after study randomization, premature delivery, and delivery outside the health care system with no access to birth information. The pregnant women’s mean age was 28 years (range: 14–44), mean body mass index was 24kg/m^2^ (range: 14_·_2 to 45_·_0) and mean GA at entry was 13 weeks (range: 6_·_7 to 18 weeks). The mean (SD) PMPD were similar– 22.9 and 22.2 in the intervention and control groups respectively, however they were significantly different (p = 0.038).

**Table 1:** Baseline demographic and clinical characteristics, by intervention arm.

Variable	Intervention (n = 162)	Control (n = 162)
**Mother’s age, years**		
Mean (SD)	28·0 (6·1)	27·9 (5·4)
Range	15–44	14–42
Missing	10	12
**Number of children**		
None	41 (25·5%)	42 (25·9%)
One or two	72 (44·7%)	71 (43·8%)
Three or four	37 (23·0%)	45 (27·8%)
More then 4	11 (6·8%)	4 (2·5%)
Missing	1	0
**Marital Status**		
Single	17 (10·6%)	7 (4·3%)
Married	143 (88·8%)	155 (95·7%)
Separated	1 (0·6%)	0 (0·0%)
Missing	1	0
**Mother’s BMI (kg/m** ^**2**^ **)**		
Mean (SD)	23·2, 4·2	24·7 (5·3)
Range	14·2–36·2	17·1–45·0
Missing	10	12
**Gestational age at entry, week**		
Mean (SD)	12·9 (3·0)	13·1 (3·0)
Range	6·7–18·0	7·1–45·0
Missing	3	12
**Educational level**		
None	51 (31·7%)	58 (35·8%)
Primary	16 (9·9%)	17 (10·5%)
Secondary	68 (42·2%)	60 (37·0%)
High/Polytechnic	17 (10·6%)	12 (7·4%)
University	0 (0·0%)	2 (1·2%)
Missing	1	0
**Household 72-hr PM**_**2.5**_ **exposure n (sd)**		
1^st^ Quartile (0–27·55)	28 (17·3)	24 (14·8)
2nd Quartile = 27·55–42·17	26 (16·1)	26 (16·1)
3^rd^ Quartile = 42·17–66·89	27 (16·7)	25 (15·4)
4^th^ Quartile = 66·89	24 (14·8)	27 (16·7)
Missing	57 (35·2)	60 (37·0)

BMI = body mass index.

### Association of PMPD with fetal biometric parameters and birth anthropometric measures

Univariate analysis (Pearson’s correlation analysis) demonstrated significant correlations (p<0_·_05) between PMPD scores and fetal biometric parameters (UEFW, HC, AC, FL, BPD). Though the correlation was significant for BL (p < 0_·_05), no significant association was observed between PMPD scores and BW, IUGR and GA. Table 2 presents the univariate results of the association between PMPD and the various endpoints (fetal biometric parameters, birth anthropometric measures, IUGR and GA). Table 3 presents results of the multivariate analysis with (a) and without (b) child’s gender included in the model. After controlling for all potential variables and including child’s gender in the model, BW, BL and IUGR were found to be significantly associated with PMPD scores. With child’s gender excluded from the model, UEFW, HC, AC, FL, BPD and BW had robust associations with PMPD. The mediation analysis revealed that the proportion of the outcomes (fetal biometrics, birth anthropometrics, IUGR and GA), which can be explained via PMPD by groups (intervention vs. control) after adjusting for confounding variables was 6_·_2% (0_·_062).

**Table 2:** Univariate mixed-effects linear regression analysis showing association of PMPD with various measures.

Measures	Coefficient (SE)	CI (95%)
**Fetal Biometrics**		
Ultrasound estimated fetal weight (UEFW)	40·04*** (14·72)	(11·175–68·91)
Head Circumference (HC)	0·24* (0·09)	(0·05–0·43)
Abdominal circumference (AC)	0·002 (0·002)	(-0·0018–0·008)
Femur length (FL)	0·077*(0·03)	(0·018–0·14)
Biparietal Diameter (BPD)	0·07*(0·03)	(0·01–0·12)
**Birth Anthropometrics**		
Birth Weight (BW)	10·22 (6·93)	(-3·37–23·80)
Birth Length (BL)	0·21* (0·09)	(0·04–0·38)
**Other**		
Intra-uterine Growth Restriction (IUGR)	0·002 (0·002)	(-0·0018–0·008)
Gestational Age (GA)	0·03 (0·03)	(-0·04–0·10)

Statistical significance: *, p<0·05;

**, p<0·01.

***, p<0·001.

**Table 3:** Multivariate analysis showing association between PMPD and measures.

Measures	Coefficient (SE)^a^	CI (95%)	Coefficient (SE)^b^	CI (95%)
**Fetal Biometrics**				
Ultrasound estimated fetal weight (UEFW)	26·99 (17·95)	(-8·19–62·19)	35·86* (18·18)	(0·23–71·50)
Head Circumference (HC)	0·21 (0·11)	(0·005–0·47)	0·26* (0·12)	(0·005–0·47)
Abdominal circumference (AC)	0·18 (0. ·13)	(0·07–0·44)	0·27*(0·13)	(0·01–0·53)
Femur length (FL)	0·07 (0·04)	(-0·02–0·15)	0·08* (0·043)	(-0·001–0·16)
Biparietal Diameter (BPD)	0·04 (0·03)	(-0·015–0·11)	0·07*(0·03)	(-0·001–0·1)
**Birth Anthropometrics**				
Birth Weight (BW)	21·03*(8·32)	(6·60–39·99)	20·38*(8·52)	(6·60–39·99)
Birth Length (BL)	0·21* (0·11)	(0·003–0·43)	0·20 (0·11)	(0·007–0·45)
**Other**				
Intra-uterine Growth Restriction (IUGR)	0·008** (0·003)	(0·002–0·01)	0·006 (0·003)	(-0·0007–0·012)
Gestational Age (GA)	-0·01 (0·02)	(-0·07–0·047)	-0·01 (0·03)	(-0·07–0·04)

Statistical significance: *, p<0·05;

**, p<0·01.

***, p<0·001;

^a^, Multivariate analysis with gender;

^b^, Multivariate analysis without gender.

Adjusted for: Number of children, age in years, educational level, BMI, Mothers serum biomarkers on nutritional status: Folic acid, albumin, pre albumin, retinol binding-protein (RBP), Malaria status, frequency of cooking, and kitchen location.

### Association between levels of PM_2.5_ and PMPD

Table 4 presents the results of univariate and multivariate analyses of the relationship between PM_2.5_ exposure and PMPD. We found no association between the levels of PM_2.5_ exposure and PMPD with either the univariate (not shown here) or multivariate analysis. The lack of trend in the coefficients with higher frequency of cooking per day suggests that the single significantly lower coefficient of PM2.5 exposure associated with four times/day cooking frequency should not be seriously considered.

**Table 4:** Univariate and multivariate analyses of association between PM_2_·_5_ exposure and PMPD.

		Model 1		Model 2	
	Coefficient (SE)		CI (95%)	Coefficient (SE)	CI (95%)
**Household PM**_**2.5**_ (μ) (ref = 0–27·55)					
1^st^ Quartile = 27·55–42·17		-0·41 (0·35)	(-1·11–0·27)	-0·56 (0·38)	(-1·32–0·20)
2^nd^ Quartile = 42·17–66·89		-0·04 (0·35)	(-0·74–0·65)	-0·004 (0·38)	(-0·76–0·75)
3^rd^ Quartile = 66·89		-0·03 (0·35)	(-0·74–0·65)	-0·24 (0·37)	(-0·98 –-0·49)
**Number of Children** (ref = 1)					
2				-0·35 (0·37)	(-1·07–0·37)
3				-0·30 (0·45)	(-1·18–0·57)
4				-1·87* (0·75)	(-3·34 –-0·40)
**Cooking times/day** (ref **=** Once/twice)					
Three times/day				-0·63 (0·42)	(-1·47–0·20)
Four times/day				-1·30** (0·46)	(-2·21 –-0·38)
Five times/day				0·17 (0·64)	(-1·09–1·45)
**Cooking Source** (ref = Indoor)					
Outside				-0·71 (0·38)	(-1·46–0·03)

Statistical significance: *, p<0·05;

**, p<0·01.

***, p<0·001; Model 1: Household PM_2_·_5_.

Model 2: Model 1 + number of children, child gender, marital status, mother’s age in years, education level, BMI, cooking times per day, cooking space mother’s malaria status, Mothers serum biomarkers on nutritional status, Folic acid, albumin, pre albumin, retinol binding-protein (RBP).

### Association between PMPD and groups (intervention and control)

Table 5 presents the results of univariate and multivariate analyses of the association of PMPD with types of fuels that the pregnant women cooked with during the study. The univariate analysis (Model 1) showed that women in the control group (firewood/kerosene users) experienced significantly higher distress levels (p = 0_·_005 for firewood; p = 0_·_01 for kerosene) compared with women in the intervention group (ethanol users). In the multivariate analysis (Model 2) in a stratified sample of the population of this study, we found that only kerosene users experienced higher distress levels (p = 0_·_05) compared with the ethanol users, while firewood users did not exhibit any such difference.

**Table 5:** Univariate and multivariate analysis of association between intervention arm and PMPD.

	Model 1		Model 2	
	Coefficient (SE)	CI (95%)	Coefficient (SE)	CI (95%)
**Intervention arm** (ref = Ethanol)				
Kerosene	-0·68* (0·28)	(1·23 –-0·13)	-0·61* (0·30)	(-1·24–0·09)
Firewood	-0·96** (0·34)	(-1·63–0·30)	-0·25 (0·45)	(-1·12–0·63)
**Number of Children** (ref = 1)				
2			-0·17(0·36)	(-0·88–0·54)
3			-0·18(0·43)	(-1·03–0·66)
4			-1·99* (0·72)	(-3·46 –-0·52)
**Cooking times/day (ref = Once/Twice)**				
Three times/day			-0·65 (0·42)	(-1·48–0·18)
Four times/day			-1·33** (0·45)	(-2·25 –-0·42)
Five times/day			0·05 (0·65)	(-1·22–1·32)
**Cooking Source** (ref = Indoor)				
Outside			-0·93* (0·27)	(-1·83–0·03)

Statistical significance: *, p<0·05;

**, p<0·01.

***, p<0·001.

Model 1: Intervention arm.

Model 2: Model 1 + number of children, gender, marital status, mother’s age, educational level, mother’s BMI, cooking times per day, cooking space, mother’s malaria status, Mothers serum biomarkers on nutritional status, Folic acid, albumin, pre albumin, retinol binding-protein (RBP).

## Discussion

To the best of our knowledge, this is the first study to report associations between PMPD and fetal biometrics, birth anthropometrics, IUGR and GA in relation to HAP exposure in sub-Saharan Africa. Our findings suggest that PMPD negatively impacts UEFW, HC, FL, BPD and BL. There was no association of PMPD with BW, AC, IUGR and estimated GA. No significant association was found between PMPD and stratified PM_2·5_ levels. The latter finding appears to contradict the significant difference in PMPD between the intervention groups in this study. A possible explanation is the difference in the units of measurement of the two variables (intervention/control versus quantitative units for PM_2.5_ converted to quartiles) and the wide variability in the raw PM_2.5_ measures. Prenatal maternal distress has been related to adverse birth outcomes [20]. However, reports from sub-Saharan Africa on this issue are few in number [7,12,21]. Ae-Ngibise K reported prenatal maternal stress negatively affected BL, BW and HC among rural Ghanaian women [12]. A team of South African researchers demonstrated associations between PMPD and fetal, infant and child developmental outcomes in a longitudinal birth cohort study [7]. Koen and colleagues also reported that maternal trauma and post-traumatic stress disorder (PTSD) adversely affected fetal growth in a birth cohort study conducted in Cape Town, South Africa [21]. Given that none of these studies reported on how household air pollution might have contributed to PMPD, which, in turn, could mediate adverse outcomes on fetal biometric and birth anthropometric parameters, our study addresses the knowledge gap in existing literature. In our view, maternal distress, air pollution exposure and pregnancy outcomes form a triad.

We investigated the association between household air pollution, maternal psychological distress, and fetal/infant growth. Psychological ill-health (depression, anxiety and stress) before, during and after pregnancy has been a constant problem in LMICs, including Nigeria, but one that remains ignored due to stigma associated with such disorders [22]. Given the significant global health importance of this problem, it is necessary to undertake larger prospective RCTs because the outcomes have implications for later life child development. Prenatal exposure to environmental contaminants has been reported to result in early childhood diseases, including asthma, wheezing, respiratory infections, altered immune defense, diabetes and heart disease [23]. Similarly, reduced fetal biometrics and birth anthropometrics have been linked to later-life impairments, both physical and neurocognitive. For example, smaller head circumference may increase the likelihood of childhood allergy and cognitive impairment in young adults [24,25]. We, further, investigated the effect of child’s gender on PMPD score by multivariate analysis with and without child’s gender included in the model. After controlling for all potential variables and including child’s gender in the model, BW, BL and IUGR were found to be significantly associated with PMPD scores. With child’s gender excluded from the model, UEFW, HC, AC, FL, BPD and BW had robust associations with PMPD. Ae-Ngibise et al., found a similar association where female newborns were found to be more vulnerable to birth outcome effects from prenatal maternal stress [12]. Additionally, in a small subset of stratified samples, we found that kerosene users experienced significantly higher distress levels compared with the ethanol users, which was not apparent among firewood users. This observation needs to be validated further by undertaking larger research studies, which will aim to look at this finding as the primary hypothesis.

Hence, our study has clinical and public health relevance in that it underscores the importance and impact of PMPD on fetal biometrics and birth anthropometrics in the setting of exposure to air pollution. According to Hobel and colleagues [26] perceived psychological stress associates more robustly with adverse pregnancy outcomes than measures based on lists of events judged to be ‘likely stressors’ for most people. The focus of our study on PMDP based on the women’s own experience of psychological distress, participation of non-smoking women, and the prospective study design of the parent RCT are strengths of the study. This is also the first study from Nigeria to explore the association of PMPD with fetal biometrics in relation to personal exposure to HAP. There are some limitations of this study. First, an abridged version of the SF-12v2^TM^ HRQOL questionnaire was used to assess PMPD and was not validated. In addition, the SF-12 v2^TM^ might not have captured all aspects of psychological distress but we believe the scale is a useful proxy measure. A validation study comparing psychometric properties of our abridged version to those of the full SF-12v2^TM^ questionnaire would be helpful. In this study, we analyzed secondary data from an original RCT, and no formal sample size or power calculations were done, thus the possibility of inadequate power to detect some associations cannot be completely ruled out. As earlier mentioned, recruitment and randomization occurred between 16 and 19 weeks, so 156 days was as long as we could observe the pregnant women during the study. Furthermore, although we explored differences in PMPD between the intervention and control groups, this was a secondary objective. Studies with much larger enrolment of participants are required to further explore the complex relationships between PMPD, HAP and fetal growth parameters. The PMPD levels before pregnancy was not collected.

## Conclusions

PMPD was an independent mediator of adverse fetal biometric parameters in pregnant women, who were exposed to HAP from burning of firewood/kerosene. Formulating preventative measures to alleviate maternal distress during pregnancy and lessening exposure to HAP is important for public health. It is also necessary to integrate consideration of psychological health matters into reproductive health policies, especially in LMICs.

## Supporting information

S1 Data (CSV)
